# Privacy Concerns Among Older Adults Using Voice Assistant Systems

**DOI:** 10.1093/geroni/igab046.1023

**Published:** 2021-12-17

**Authors:** Hillary Spangler, Tiffany Driesse, Robert Roth, Xiaohui Liang, David Kotz, John Batsis

**Affiliations:** 1 UNC Hospitals, Chapel Hill, North Carolina, United States; 2 UNC School of Medicine, Chapel Hill, North Carolina, United States; 3 Dartmouth-Hitchcock, Lebanon, New Hampshire, United States; 4 University of Massachusetts Boston, Boston, Massachusetts, United States; 5 Dartmouth College, Hanover, New Hampshire, United States; 6 University of North Carolina at Chapel Hill, Chapel Hill, North Carolina, United States

## Abstract

Voice Assistant Systems (VAS) are software platforms that complete various tasks using voice commands (e.g., Amazon Alexa), with increasing usage by older adults. It is unknown whether older adults have significant privacy concerns with VAS. 55 participants were evaluated from ambulatory practice sites for a study on VAS detection of early cognitive decline. The mean age was 73.3±5.6 years, 58% female, 93% white, and 53% had mild cognitive impairment. Privacy concerns were assessed via Likert-based surveys. Participants believed data was used with consent (71%) and stored properly (67%); however, 71% wanted new privacy regulations, 43% were comfortable with daily activity monitoring, and 85% thought the data needs to be highly protected. Qualitative themes included “listening-in”, “tracking”, and unwanted sharing of information. Findings suggest that older adults do not have significant privacy concerns for VAS use, but requested additional regulations. Future research can compare VAS privacy concerns between age groups.

